# Determining the Best Treatment for Coronal Angular Deformity of the Knee Joint in Growing Children: A Decision Analysis

**DOI:** 10.1155/2014/603432

**Published:** 2014-09-03

**Authors:** Ki Hyuk Sung, Chin Youb Chung, Kyoung Min Lee, Seung Yeol Lee, In Ho Choi, Tae-Joon Cho, Won Joon Yoo, Moon Seok Park

**Affiliations:** ^1^Department of Orthopaedic Surgery, Myongji Hospital, 55 Hwasu-ro, 14 Beon-gil, Deokyang-gu, Goyang, Kyungki 412-826, Republic of Korea; ^2^Department of Orthopaedic Surgery, Seoul National University Bundang Hospital, 300 Gumi-Dong, Bundang-gu, Sungnam, Kyungki 463-707, Republic of Korea; ^3^Department of Orthopaedic Surgery, Seoul National University Children's Hospital, 101 Daehak-ro, Jongno-gu, Seoul 110-744, Republic of Korea

## Abstract

This study aimed to determine the best treatment modality for coronal angular deformity of the knee joint in growing children using decision analysis. A decision tree was created to evaluate 3 treatment modalities for coronal angular deformity in growing children: temporary hemiepiphysiodesis using staples, percutaneous screws, or a tension band plate. A decision analysis model was constructed containing the final outcome score, probability of metal failure, and incomplete correction of deformity. The final outcome was defined as health-related quality of life and was used as a utility in the decision tree. The probabilities associated with each case were obtained by literature review, and health-related quality of life was evaluated by a questionnaire completed by 25 pediatric orthopedic experts. Our decision analysis model favored temporary hemiepiphysiodesis using a tension band plate over temporary hemiepiphysiodesis using percutaneous screws or stapling, with utilities of 0.969, 0.957, and 0.962, respectively. One-way sensitivity analysis showed that hemiepiphysiodesis using a tension band plate was better than temporary hemiepiphysiodesis using percutaneous screws, when the overall complication rate of hemiepiphysiodesis using a tension band plate was lower than 15.7%. Two-way sensitivity analysis showed that hemiepiphysiodesis using a tension band plate was more beneficial than temporary hemiepiphysiodesis using percutaneous screws.

## 1. Introduction

Coronal angular deformity of the lower limb is a common finding in growing children. In addition to being a cosmetic problem, it can lead to early osteoarthritis in later life because of joint overload [1]. Angular deformity can be corrected by guided growth of the physis in growing children. Permanent hemiepiphysiodesis by physeal ablation was first introduced by Phemister in 1933 [2]. For permanent fusion, accurate timing of the surgery is crucial because improper timing can lead to over- or undercorrection [3]. Therefore, temporary hemiepiphysiodesis using staples [4], percutaneous screws [5], or a tension band plate [6] is commonly used in these patients.

Although the surgical outcomes of these treatment modalities in children with coronal angular deformity have been studied, there is no consensus on the best modality, and the procedure is used based on the surgeon's preference. The 2 key points when comparing the 3 methods are the risk of metal failure and incomplete correction of deformity. Temporary hemiepiphysiodesis using percutaneous screws was reported to involve the risk of incomplete correction due to the delayed epiphysiodesis effect, while having no risk of metal failure [5, 7]. In contrast, temporary hemiepiphysiodesis using stapling has the risk of metal failure [8–12].

By eliminating selection bias, randomized controlled trials (RCTs) can objectively establish best practices, allowing physicians to provide the most effective treatment. However, RCTs require long-term followup and are associated with high costs in some areas of orthopedic research. Alternatively, decision analysis using observational studies is a logical process to identify the best option. Decision analysis, originally used in the business field, has made it possible to obtain evidence-based knowledge without performing RCTs and is a useful tool for formulating and generalizing the decision-making process [13–15].

Our aim was to determine the best treatment modality for coronal angular deformity of the knee joint in growing children using decision analysis based on the current best evidence.

## 2. Materials and Methods

The present study was exempt from institutional review board approval because it did not involve human subjects.

### 2.1. Literature Review and Determination of Decision Tree

**Figure 1:**
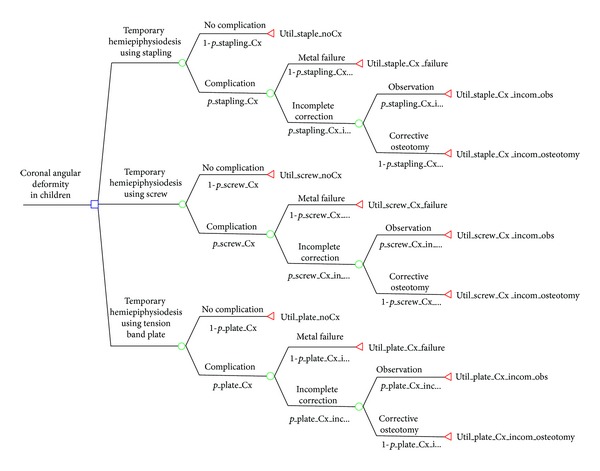
The decision analysis tree with probability and utility variables. The decision node branches into “temporary hemiepiphysiodesis using stapling,” “temporary hemiepiphysiodesis using screw,” and “temporary hemiepiphysiodesis using a tension band plate.”

Four pediatric orthopaedic surgeons with a mean of 16.3 years (range: 11 to 26 years) of orthopaedic experience were involved in literature review and determination of decision tree. Our literature review focused on the surgical outcome of coronal angular deformity of the knee joint in children after temporary hemiepiphysiodesis using stapling, percutaneous screws, or a tension band plate. We assessed 34 studies regarding temporary hemiepiphysiodesis using stapling, percutaneous screws, or a tension band plate as a treatment option for coronal angular deformity in children [5, 6, 8–12, 16–42]. A literature review revealed various surgery-related complications, including metal failure, wound infection, neuropraxia, rebound phenomenon, incomplete correction, overcorrection, and limitation of motion. Of these, we identified 2 key factors for comparing temporary hemiepiphysiodesis using stapling, percutaneous screws, and a tension band plate. These were metal failure and incomplete correction of angular deformity. According to these factors, the possible surgical outcomes and probability of each outcome were defined, and a decision tree was constructed using TreeAge Pro 2013 (TreeAge Software Inc., Williamstown, MA, USA). The 3 treatment options for children with coronal angular deformity of the lower limb were temporary hemiepiphysiodesis using stapling, percutaneous screws, or a tension band plate; at this point, the root node divided into 3 arms. Each option then branched into chance nodes and terminated at an endpoint clinical outcome, termed “utility,” in the decision tree. Each branching point indicated the probability of each event (Figure 1).

### 2.2. Branch

Each treatment modality branched into “No complication” and “Complication,” according to the presence of complications. With complications, the chance node branched into “metal failure” and “incomplete correction.” Incomplete correction further divided into “observation” and “corrective osteotomy” branches. Utility scores were assigned to each terminal node.

### 2.3. Event Probabilities

All baseline probability values for each node were set to the mean values reported in the literature. The baseline metal failure rate was set to 10.8% (range: 0–45.3%) [8–12, 16–19, 23, 24, 27, 31–34, 36, 41, 42] for the stapling branch, 0% (range: 0–0%) [5, 22, 25, 28, 29, 36] for the percutaneous screw branch, and 4.2% (range: 0–25.8%) [6, 11, 12, 26, 30, 31, 35, 37–42] for the tension band plate branch. The incomplete correction rate was set to 11.8% (range: 0–57.6%) [8–11, 16–21, 23, 31–34, 36, 41, 42] for the stapling branch, 14.8% (range: 5.4–33.3%) [5, 22, 28, 29, 36] for the percutaneous screw branch, and 5.0% (range: 0–12.5%) [6, 11, 26, 31, 37, 39–42] for the tension band plate branch. A literature review revealed that the rate of requirement of corrective osteotomy for incomplete correction ranged from 0% to 27.3% for the stapling branch, from 0% to 11.5% for the percutaneous screw branch, and from 0% to 10.3% for the tension band plate branch. Therefore, the baseline osteotomy rate was set to 6.4%, 5.6%, and 4.4%, respectively (Table 1).

### 2.4. Health Utilities

Utilities were measured using the responses of pediatric orthopedic surgeons to a self-administered questionnaire, which was developed to assess perceptions about the health utilities of a series of outcomes after temporary hemiepiphysiodesis for a coronal angular deformity of the lower limb in children (Appendix). 25 pediatric orthopedic surgeons with 9.0 ± 7.1 years of experience in treating coronal angular deformity in children completed the questionnaire based on their personal experiences. Respondents were asked to rank each scenario depicted in the decision tree on a scale from 0 (death) to 100 (perfect health) to score their perceptions of quality of life if faced with the event. Values were converted to a scale of 0–1.0 and then used in the decision tree (Table 2).

### 2.5. Statistical Analysis

TreeAge Pro 2013 (TreeAge Software Inc., Williamstown, MA, USA) was used to construct the decision analysis. Final expected value for quality of life was calculated using a “rollback” technique. Decision analysis is a useful systematic approach to decision making when the information is imperfect. It is based on the practical application of probability theory. It determines the optimal strategy from among a series of alternatives and seeks to identify the best alternative. In a decision tree, each branch has 2 aspects of benefit and loss and its own probability. Benefit and loss are represented by the final outcome score, termed the utility. Final outcome scores are calculated by multiplying the probabilities by the utilities. In the medical field, a final outcome score in decision analysis represents relative health status. When the score is between 0 and 1, 0 represents the worst health status possible (perhaps death) and 1 represents the best health status possible (perfect health). The final outcome score is a relative and unique value in a specific decision tree and cannot be applied to or compared with another decision tree.

### 2.6. Sensitivity Analysis

The uncertainty and stability of the decision tree model were assessed using a sensitivity analysis tool. The sensitivity analysis, which provides the threshold probabilities for each event, compensates for the uncertainty of the decision tree model. One-way sensitivity analysis was used to assess the impact of alterations in the probability of one parameter on the conclusion. The overall complication rate of temporary hemiepiphysiodesis using percutaneous screws ranged from 0.0% to 33.3%, whereas the overall complication rate of temporary hemiepiphysiodesis using a tension band plate ranged from 0% to 27.8%, according to the literature review. However, the entire possible range for each event, which was between 0% and 100%, was included. One-way sensitivity analysis was performed to determine the threshold value of the treatment modalities. The threshold value is the point of intersection of each variable. Two-way sensitivity analysis was also used to examine the combined impact of changes on the probabilities of two parameters.

## 3. Results

**Figure 2:**
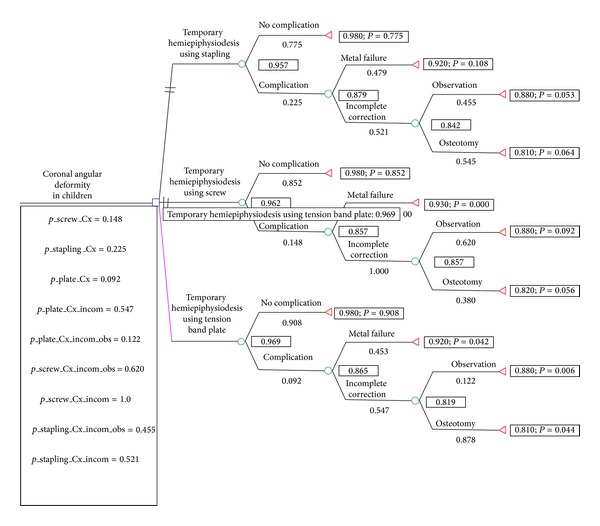
The decision analysis tree and the results of the “roll-back” process. The decision model favors temporary hemiepiphysiodesis using a tension band plate for coronal angular deformity of the knee joint in children.

The decision model showed that temporary hemiepiphysiodesis using a tension band plate was the best of the 3 treatment modalities. When performing the rollback, the expected value of temporary hemiepiphysiodesis using a tension band plate was 0.969, while those of temporary hemiepiphysiodesis using stapling and percutaneous screws were 0.957 and 0.962, respectively (Figure 2).

**Figure 3:**
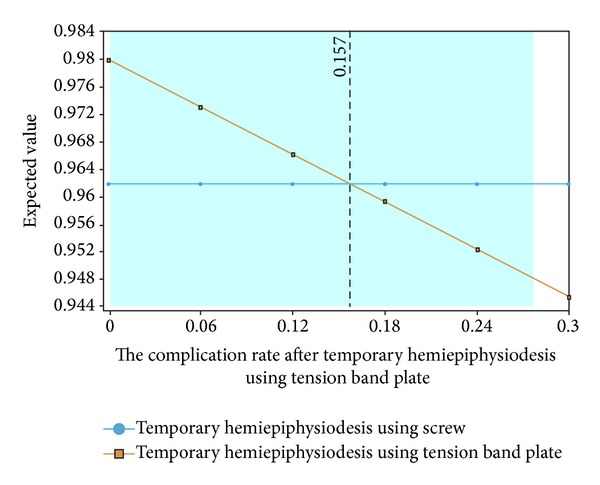
One-way sensitivity analysis on the overall complication rate of temporary hemiepiphysiodesis using a tension band plate. The decision analysis model favors temporary hemiepiphysiodesis using a tension band plate in terms of quality of life when the probability of the overall complication rate of temporary hemiepiphysiodesis using a tension band plate was lower than 15.7%.

**Figure 4:**
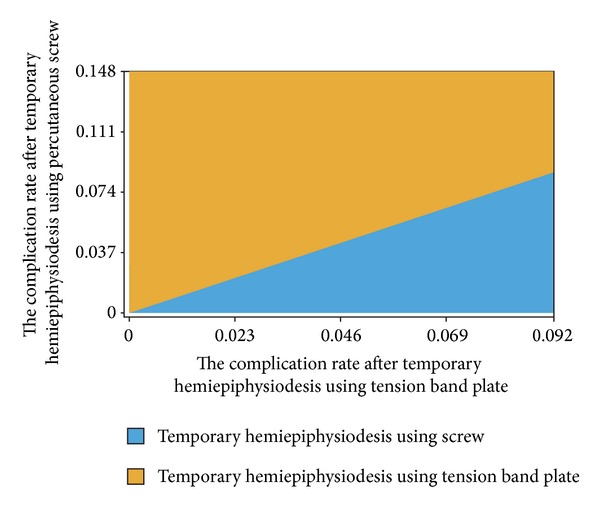
Two-way sensitivity analysis of the overall complication rate after temporary hemiepiphysiodesis using percutaneous screws and a tension band plate. This result shows preferred decision according to changes in the overall complication rate of temporary hemiepiphysiodesis using percutaneous screws and a tension band plate. Temporary hemiepiphysiodesis using a tension band plate occupied a larger area than that occupied by guided growth using percutaneous screws, suggesting that temporary hemiepiphysiodesis using a tension band plate was better than temporary hemiepiphysiodesis using percutaneous screws in the expected values.

One-way sensitivity analysis showed that the expected value of temporary hemiepiphysiodesis using a tension band plate was superior to temporary hemiepiphysiodesis using percutaneous screws when the overall complication rate of temporary hemiepiphysiodesis using a tension band plate was below 15.7% (Figure 3). The overall complication rate of temporary hemiepiphysiodesis using a tension band plate and percutaneous screws was analyzed using two-way sensitivity analysis. The results suggested that temporary hemiepiphysiodesis using a tension band plate was better than temporary hemiepiphysiodesis using percutaneous screws in the expected values (Figure 4).

## 4. Discussion

Our decision analysis model showed that temporary hemiepiphysiodesis using a tension band plate was a better treatment modality than temporary hemiepiphysiodesis using stapling or percutaneous screws with respect to quality of life. The 2 key comparable factors considered in the analysis were metal failure and incomplete correction, based on current evidence in the relevant literature. Furthermore, sensitivity analyses showed that the derived model was relatively robust.

In 1949, Blount and Clarke [4] first introduced reversible hemiepiphysiodesis using staples, and the technique is considered to be an effective and safe method to achieve angular correction [9, 10, 19–21, 32, 34]. However, several studies have shown that the procedure can be associated with premature physeal closure, breakage or migration, and difficult removal [5, 6, 12, 17–19]. In 1998, Métaizeau et al. [5] described a new technique for percutaneous epiphysiodesis using transphyseal screws. This method has been widely used because of its many advantages such as effective angular correction, minimal morbidity, short hospital stay, early rehabilitation, fewer complications, and good cosmesis [5, 22, 25, 28, 29, 36]. Recently, Stevens [6] proposed a new device, the eight-plate, consisting of an extraperiosteal 2-hole plate and screws; this device serves as a tension band. A number of studies have reported a favorable outcome with the eight-plate method in terms of angular correction, speed of correction, and minimal hardware problems [12, 26, 31, 35, 37, 38].

Several studies have compared temporary hemiepiphysiodesis using physeal stapling with temporary hemiepiphysiodesis using a tension band plate in children with coronal angular deformity [11, 12, 31, 41, 42]. They concluded these modalities were equally effective with respect to rate of correction and complications. However, temporary hemiepiphysiodesis with a tension band plate was preferable because of the precision of the surgical technique, short surgical time, and less hardware failure. Two previous studies have compared percutaneous screws with physeal stapling [28, 36]. They concluded that temporary hemiepiphysiodesis using percutaneous screws was as effective as hemiepiphyseal stapling in terms of angular deformity correction and was less invasive with a better cosmetic result. No metal failure has been reported in previous studies. However, Ilharreborde et al. found a delayed epiphysiodesis effect with percutaneous screws, and they recommend using the tension band plate on the tibial side because of the high rate of screw-related pain and difficulties in screw removal [7].

To provide a decision guide for selecting a treatment modality, a study comparing the 3 methods, especially an RCT, is needed. However, it would be very difficult to perform an RCT because of the long-term follow-up period required and potential high cost. A recent RCT compared temporary hemiepiphysiodesis using a tension band plate with temporary hemiepiphysiodesis using stapling for idiopathic genu valgum [42]. That study found no significant differences between the two techniques because of a small sample size of 10 children in each group. Therefore, we performed a decision analysis based on current evidence in the relevant literature to investigate the best treatment modality for coronal angular deformity in growing children. In the present study, quality of life was used to unify the clinical outcomes of metal failure and incomplete correction of deformity, which are the 2 key points for comparing the 3 treatment modalities. Such an analysis can provide objective, clear, and intuitive results to guide the selection of a treatment modality.

Several limitations of the present study need to be addressed. First, the utilities used in this study, which were surgeon-derived, need to be validated with respect to their value to patients and the differences in clinical implications of each utility. Although patient-derived utilities might have been more meaningful clinically, these patients have great difficulty completing a questionnaire about the quality of life after surgery. To overcome this shortcoming, we utilized surgeon-derived utility values by surveying the opinions of experts, as described elsewhere [43–45]. Second, decision analysis depends on varying the probabilities of specific events over a continuum. To address this limitation, we undertook sensitivity analyses over a broad range of clinically pertinent values, and we performed sensitivity analysis, which demonstrated the relative stability of our decision model. Third, there were various surgery-related complications, including metal failure, wound infection, neuropraxia, rebound phenomenon, permanent physeal closure, difficulty in implant removal, incomplete correction, and limitation of motion. However, the incidence of the permanent physeal closure was very rare, and the others were not a significant complication considered as primary variable in decision analysis. Therefore, our model included only two key factors, such as metal failure and incomplete correction. Fourth, the expected values of 3 treatment modalities were quite similar. Papers regarding staple hemiepiphysiodesis were older than those regarding screw or plate hemiepiphysiodesis and the higher failure rate might be reported in the paper regarding staple hemiepiphysiodesis due to use without clear indications. Therefore, the expected value of stale epiphysiodesis might be underestimated. However, recent papers regarding staple hemiepiphysiodesis reported the higher failure rate than old ones.

Despite these limitations, the present study indicates that temporary hemiepiphysiodesis using a tension band plate may provide a better quality of life than temporary hemiepiphysiodesis using percutaneous screws or stapling for children with coronal angular deformity of the knee joint.

## Figures and Tables

**Table 1:** Definition of variables with baseline probabilities in the decision tree.

Variable	Tree definition	Baseline	Range	Studies
Rate of outcomes after temporary hemiepiphysiodesis using stapling	*P*_staple_Cx	0.225	0.000~0.667	Pistevos and Duckworth [16], Zuege et al. [17], Fraser et al. [18], Mielke and Stevens [19], Stevens et al. [20], Raab et al. [8], Degreef et al. [21], Westberry et al. [9], Park et al. [10], Eidelman and D'Agostino [23], Stevens and Pease [11], Novais and Stevens [24], Castañeda et al. [27], Stevens and Klatt [12], Wiemann et al. [31], Courvoisier et al. [32], Bushnell et al. [33], Cho et al. [34], Shin et al. [36], Jelinek et al. [41], Gottliebsen et al. [42]
	*P*_staple_metal failure	0.108	0.000~0.453	
	*P*_staple_incomplete correction	0.118	0.000~0.576	
	*P*_staple_incomplete correction_observation	0.053	0.000~0.303	
	*P*_staple_incomplete correction_osteotomy	0.064	0.000~0.273	

Rate of outcomes after temporary hemiepiphysiodesis using screw	*P*_screw_Cx	0.148	0.000~0.333	Métaizeau et al. [5], Nouh and Kuo [22], Khoury et al. [25], Brauwer and Moens [28], Mesa and Yamhure [29], Shin et al. [36]
	*P*_screw_metal failure	0.000	0.000~0.000	
	*P*_screw_incomplete correction	0.148	0.054~0.333	
	*P*_screw_incomplete correction_observation	0.092	0.000~0.333	
	*P*_screw_incomplete correction_osteotomy	0.056	0.000~0.115	

Rate of outcomes after temporary hemiepiphysiodesis using tension band plate	*P*_plate_Cx	0.092	0.000~0.278	Stevens and Pease [11], Stevens [6], Stevens and Klatt [12], Burghardt et al. [26], Wiemann et al. [31], Schroerlucke et al. [30], Burghardt and Herzenberg [37], Ballal et al. [35], Guzman et al. [38], Boero et al. [39], Scott [40], Jelinek et al. [41], Gottliebsen et al. [42]
	*P*_plate_metal failure	0.042	0.000~0.258	
	*P*_plate_incomplete correction	0.050	0.000~0.125	
	*P*_plate_incomplete correction_observation	0.006	0.000~0.056	
	*P*_plate_incomplete correction_osteotomy	0.044	0.000~0.103	

*P*: probability; Cx: complication.

**Table 2:** Estimated utility scores used in the decision tree.

Variable	Tree definition	Utility score
Temporary hemiepiphysiodesis using stapling	Util_staple_No Cx	0.98
	Util_staple_Cx_metal failure	0.92
	Util_staple_Cx_incomplete correction_observation	0.88
	Util_staple_Cx_incomplete correction_osteotomy	0.81

Temporary hemiepiphysiodesis using screw	Util_screw_No Cx	0.98
	Util_screw_Cx_metal failure	0.93
	Util_screw_Cx_incomplete correction_observation	0.88
	Util_screw_Cx_incomplete correction_osteotomy	0.82

Temporary hemiepiphysiodesis using tension band plate	Util_plate_No Cx	0.98
	Util_plate_Cx_metal failure	0.92
	Util_plate_Cx_incomplete correction_observation	0.88
	Util_plate_Cx_incomplete correction_osteotomy	0.81

Util: utility; Cx: complication.

**Table 3:** 

	Estimated QoL
Temporary hemiepiphysiodesis using stapling	
No complication	
Metal failure	
Incomplete correction with observation	
Incomplete correction with osteotomy	
Temporary hemiepiphysiodesis using percutaneous screw	
No complication	
Metal failure	
Incomplete correction with observation	
Incomplete correction with osteotomy	
Temporary hemiepiphysiodesis using a tension band plate	
No complication	
Metal failure	
Incomplete correction with observation	
Incomplete correction with osteotomy	

## Appendix

# How long have you been treating coronal angular deformity of the knee joint in children?

_—_ years

This is an assessment of the treatment of coronal angular deformity of the knee joint in growing children. Please complete Table 3 by providing a quality of life (QoL) score for each situation after treatment of coronal angular deformity of the knee joint in children on a scale of 0 to 100 points (with perfect QoL score being 100 and death being 0). (see Table 3).
